# Response: Commentary: Cortical responses to salient nociceptive and not nociceptive stimuli in vegetative and minimal conscious state

**DOI:** 10.3389/fnhum.2016.00012

**Published:** 2016-01-29

**Authors:** Marina de Tommaso

**Affiliations:** Neurophysiopathology of Pain, Basic Medical Sciences, Neuroscience and Sensory System (SMBNOS) Department, Bari Aldo Moro UniversityBari, Italy

**Keywords:** consciousness, pain measurement, laser evoked potentials, EEG, gamma band

In their interesting comments, Naro and Calabrò (2015) pointed out the reliability and potential utility of Laser Evoked Potentials (LEPs) examination in the assessment of residual capacities of patients with Disorder of Consciousness. The main issue of their letter, regarded the possibility to *complement* LEPs assessment by means of a novel analysis focusing on EEG oscillating activities in high frequency-gamma-range, in accord to recent studies (Gross et al., 2007; Zhang et al., 2012). These comments arise from the valuable experience of the same group, who recently published interesting studies on this topic. One study (De Salvo et al., 2015) evaluated LEPs in a well-selected cohort of 13 vegetative (VS) and 10 minimal conscious state (MCI) patients. The correlation they found between LEPs latency prolongation and clinical features and particularly Coma Recovery State, led to quite different interpretation as compared to our results (de Tommaso et al., 2013, 2015). In fact, while we observed LEPs presence even in VS patients independently from the deterioration of other cognitive functions, De Salvo et al. (2015) suggested that cortical potentials by nociceptive stimuli might be lost in severe disorder of consciousness and that their preservation may be a sign of possible clinical recovery. This question opens other important issues concerning the potential preservation of pain sensitivity in all patients with autonomous vegetative control, and the general significance of pain in human behavior. In fact, in the case we accept the thesis that the cortical response against the nociceptive stimuli is preserved in the vegetative state, as LEPs presence may suggest, clinicians should consider that patients might feel pain also in absence of a clear motor behavior expressing suffering. Different results across studies may be subtended by the presence of artifacts and noise, diverse montages, and stimulus characteristics, which may justify the use of more conservative, reliable and objective analysis methods. We previously attempted to use a visual analysis of LEPs recorded by multichannel montage in single patients, in order to improve the reliability of cortical potentials by means of individual MRI supported scalp model (de Tommaso et al., 2013). In a more recent study, we compared LEPs with multimodal non-nociceptive evoked responses, to confirm our previous impression that the cortical *response* to pain is preserved in the vegetative state independently from the potentials related to other stimuli modalities (de Tommaso et al., 2015). These methods need to be further improved, in order to obtain a good assessment of pain signals conduction and cortical responsiveness in such difficult patients. The possibility to compute EEG high frequencies changes to prove the residual capacity of cortical areas to perceive the salience of nociceptive stimuli in VS and MCI patients (Zhang et al., 2012) is an intriguing challenge for further studies. In fact, fast EEG frequencies recorded in resting state were indicated as a good prognostic marker for recovery and survival (Sitt et al., 2014), so the possible preservation of cortical functions electively dedicated to pain processing may also indicate the residual ability of brain in fast oscillations. The analysis of gamma activities related to painful stimuli may contribute to dissect the cortical resources devoted to the generic salience of the stimuli, from the cortical response specific to nociceptive inputs, which may characterize the less compromised patients (Zhang et al., 2012). More recently, Naro et al. (2015), reported that rTMS over the anterior cingulate was able to modulate gamma-oscillations even in patients with severe disorder of consciousness, who may perceive pain also in absence of evident motor reaction. In this sense, we completely agree with the authors that such analysis may improve the detection of a cortical response specific for painful stimuli also in severe brain injuries, though caution should be used in the results interpretation. First of all, this pattern needs to be fully validated in normal subjects, before being applied to patients with severe brain injury, as to date there is still not definitive evidence that they reflect “pain” rather than the processing of salient stimuli. A not less important question arises in the complex processes of functional re-organization which may completely change the significance of brain rhythms oscillations (Sitt et al., 2014). In addition, evoked responses and EEG changes by laser stimuli, may only indicate that nociceptive stimuli activate cortex, but they not explain the complexity of pain processing. Considering that pain is a pivotal component of patients suffering, the best understanding of possible pain feeling also in absence of a reliable behavioral motor reaction, could be addressed by an integrated clinical and neurophysiological approach. Clinical scales, based on the careful patients observation and application of new clinical scores (Corbett et al., 2014), together with the employment of standard neurophysiological assessment including laser evoked responses, completed by more sophisticated methods of EEG analysis, need to be applied in as larger as possible patients cohorts in longitudinal and multicenter study designs. An integrated clinical and neurophysiological assessment of the perception, reaction and cortical processing of pain may represent the only way to increase the significance of these preliminary results and resolve the question if pain feeling is essential for human life and should be taken into consideration also in apparent absence of awareness.

## Author contributions

The author confirms being the sole contributor of this work and approved it for publication.

### Conflict of interest statement

The author declares that the research was conducted in the absence of any commercial or financial relationships that could be construed as a potential conflict of interest.
